# Effects of species mixing on maximum size–density relationships in Chinese fir (*Cunninghamia lanceolata* (Lamb.) Hook.)-dominated mixed forests converted from even-aged pure stands

**DOI:** 10.3389/fpls.2024.1342307

**Published:** 2024-04-03

**Authors:** Yuanyuan Han, Baichang Wang, Honggang Sun

**Affiliations:** ^1^ Research Institute of Subtropical Forestry of Chinese Academy of Forestry, Hangzhou, Zhejiang, China; ^2^ Nanjing Forestry University, Nanjing, China; ^3^ East China Inventory and Planning Institute, National Forestry and Grassland Administration, Hangzhou, China; ^4^ Research Institute of Fast-growing Trees, Chinese Academy of Forestry, State Key Laboratory of Efficient Production of Forest Resources, Hangzhou, China

**Keywords:** Chinese fir-dominated mixed forests, maximum size-density relationships, mixing proportion, latitude, site conditions

## Abstract

**Introduction:**

Density management is a key silvicultural tool in management programs that enhances compositional and structural diversity and hence forest growth during the conversion of even-aged pure stands into mixed forests.

**Methods:**

To determine the optimum stand density, a model of maximum size–density relationships was employed to explore the relationship of the self-thinning trajectory with growth, species mixing, latitude, and site conditions during the transition of even-aged pure Chinese fir stands to Chinese fir-dominated mixed forests using stochastic frontier analysis. Data were obtained from a total of 591 permanent plots located in Fujian, Jiangxi, Zhejiang, and Anhui provinces in southern China.

**Results:**

The results showed that (1) the slope of the maximum size–density relationship of Chinese fir-dominated mixed forests increased and plateaued over time; (2) the slope of the maximum size–density relationship of Chinese fir-dominated mixed forests did not deviate from Reineke’s assumed universal slope of -1.605; and (3) mixing proportion had a positive effect on maximum size–density relationships, and latitude and site conditions had the opposite effect on maximum size–density relationships.

**Conclusions:**

Our findings will provide valuable guidance for the forest management of areas in which even-aged pure stands are being converted to mixed forests (i.e., when broadleaved tree species are planted after thinning to improve overall stand density and promote stand growth).

## 1 Introduction

As plants grow in size, their demands for resources and growing space increase. If resources become increasingly scarce for all living individuals in a pure stand with growth, the death of small trees can occur because of asymmetric competition between large and small trees (Adler, 1996; Sackville Hamilton et al., 1995; Berger and Hildenbrandt, 2003; Sun et al., 2011). Eventually, forests reach the maximum stand density that can be tolerated by the average individual size, and the size–density curve approximates a straight line on a double logarithmic scale (Sampson and Smith, 1993; Stoll et al., 2002; Frank et al., 2018).

In even-aged pure stands, both the slope and intercept of the maximum size–density relationship can remain unchanged (Reineke, 1933), one of them can change (Bi, 2001; Pretzsch and Biber, 2005), or both of them can change (Zeide, 1987; Weiskittel et al., 2009) as stands mature, and this depends on the tree species (Harper, 1977), site conditions (Bi, 2001), nutrient availability (Morris, 2002), climate (DeBell et al., 1989), and species interactions (Fleischbein et al., 2005; Comeau et al., 2010). However, most studies have shown that the self-thinning slope is close to the theoretical value -1.605 (*N∞d^-1.605^*), which indicates that the size of trees increases as the number of trees per hectare decreases (e.g., Bi, 2001; Sun et al., 2011; Kimsey et al., 2019). Maximum size–density trajectories in mixed forests can be altered by species mixing (Pretzsch et al., 2012), site conditions (Harms et al., 2000; Pittman and Turnblom, 2003; VanderSchaaf and Burkhart, 2008), and latitude, including precipitation and atmospheric temperature (Ducey et al., 2017; Kweon and Comeau, 2017). The self-thinning relationship of mixed forests can shift upwards because the packing density is higher in interspecific neighborhoods than in intraspecific neighborhoods. Compared with pure stands, mixed-species forests with the same seedling age have size–density relationships with flatter slopes because of their higher stand densities, which improves resource availability and alleviates intraspecific and interspecific competition (Kelty, 1992; Forrester et al., 2006; Pretzsch et al., 2012). For example, an analysis of 432 triplets of common tree species mixtures derived from long-term experiments in Central Europe has shown that mixed forests are 15% denser on average than the weighted mean of the neighboring pure stands, and the slope of the self-thinning relationship deviated from -1.605 (Pretzsch and Biber, 2005).

The timber rotation period of pure stands is shorter than that of the nutrient cycle period from one generation to the next; there is thus a lasting decline in stand productivity in areas with low water and nutrient supplies (Wang et al., 2022). Similar to other pure timber stands, especially *Eucalyptus* stands in Brazil, *Poplar* stands in Canada, *Radiata* pine stands in New Zealand, and Chinese fir stands in China, sustainable forest management is hindered by soil degradation and fierce intraspecific competition (Liu et al., 2018). Improving natural regeneration in canopy gaps and soil conditions to reduce intraspecific and interspecific competition is an ecologically and economically effective approach for addressing this problem (Weiskittel et al., 2009; Richards et al., 2010).

The stand productivity of mixed-species forests can be determined through analysis of two-tree-species mixtures with seedlings of the same age at the same planted phase. However, in most conifer plantations, including Chinese fir stands, pure stands have been converted to mixed broad-leaved Chinese fir forests by removing small trees and retaining dominant ones, which facilitates the growth of natural broad-leaved seedlings in the canopy gaps. Chinese fir is the most important timber tree species in terms of area both within and outside of China; it comprises approximately 24% and 6.1% of forest plantations in China and worldwide, respectively (Liu et al., 2018; Yang and Burkhart, 2018). Clarifying maximum size–density relationships for mixtures of dominant planted tree species and natural broad-leaved tree species can provide valuable information for stand density management.

The objectives of this paper were to determine (1) whether the maximum size–density relationships shifted upwards or downwards in Chinese fir-dominated mixed forests compared with the *N∞d^-1.605^* self-thinning trajectory; (2) how tree mixing affects maximum size–density relationships; and (3) whether maximum size-density relationships in mixed-species forests are affected by mixing proportion, site conditions, and latitude.

## 2 Materials and methods

### 2.1 Study area and data description

**Figure 1:**
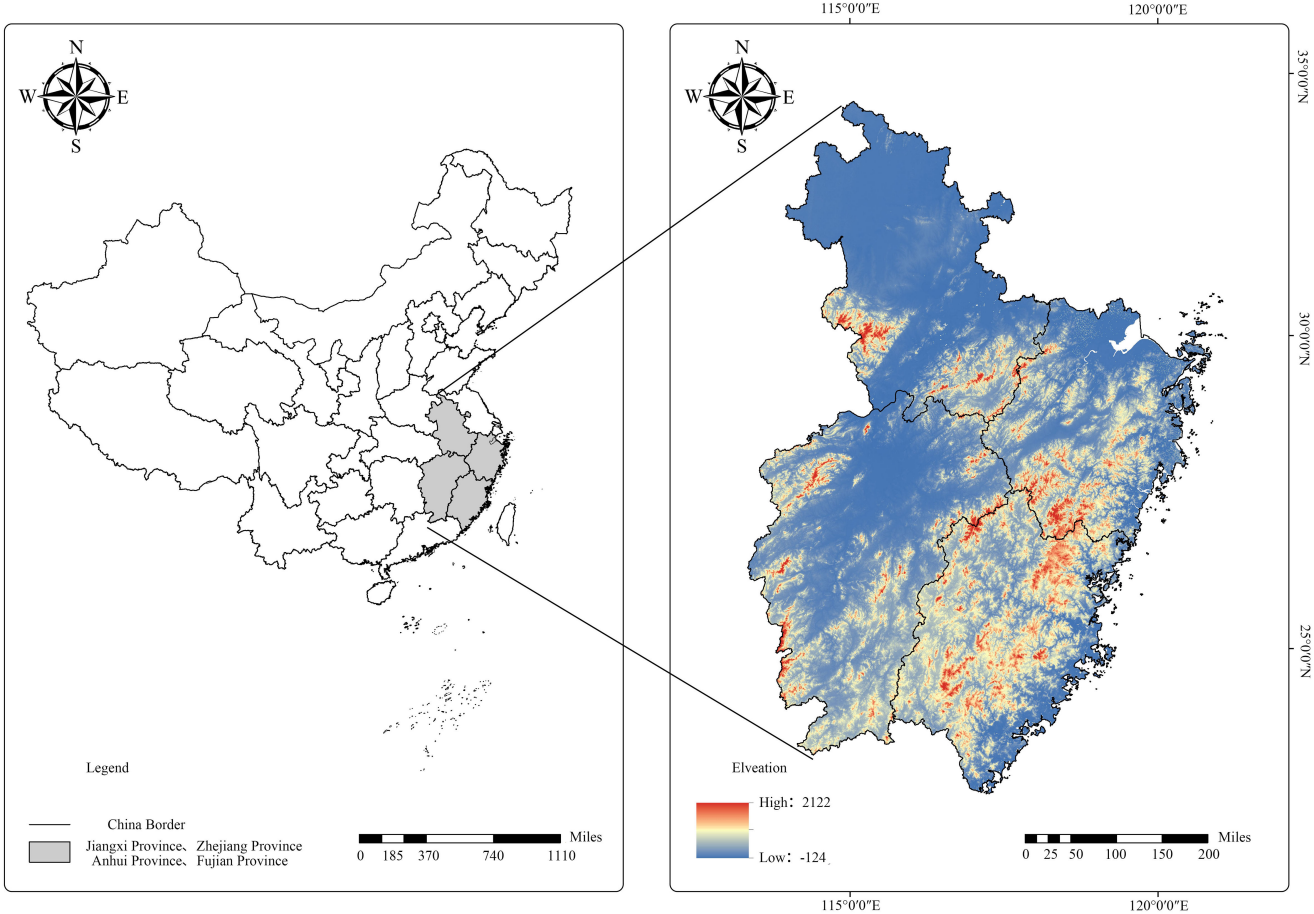
Chinese fir forest study sites in China.

We analyzed the self-thinning relationship of Chinese fir-dominated mixed forests using National Forest Inventory (NFI) data for mainland China. Data for every 5 years of observations from 1991 to 2016 were obtained for Fujian, Jiangxi, Zhejiang, and Anhui provinces, which included 61.1% of areas with Chinese fir in China (**Table 1**; **Figure 1**).

**Table 1:** Site characteristics of National Forest Inventory (NFI) plots in Fujian, Jiangxi, Zhejiang, and Anhui provinces used in this study.

Variables	Fujian province	Jiangxi province	Zhejiang province	Anhui province
Altitude(m)	220-990	20-850	250-1000	10-1020
Maximum Temperature (°C)	40	39	41	40
Annual Mean Temperature (°C)	18-26	16-23	15-23	13-22
Minimum Temperature (°C)	-1	-7	-10	-10
Precipitation (mm)	1477	1518	1567	978
Soil Type	Red soil, Yellow soil	Red soil	Red soil, Yellow soil	Yellow brown soil, Yellow soil

**Figure 2:**
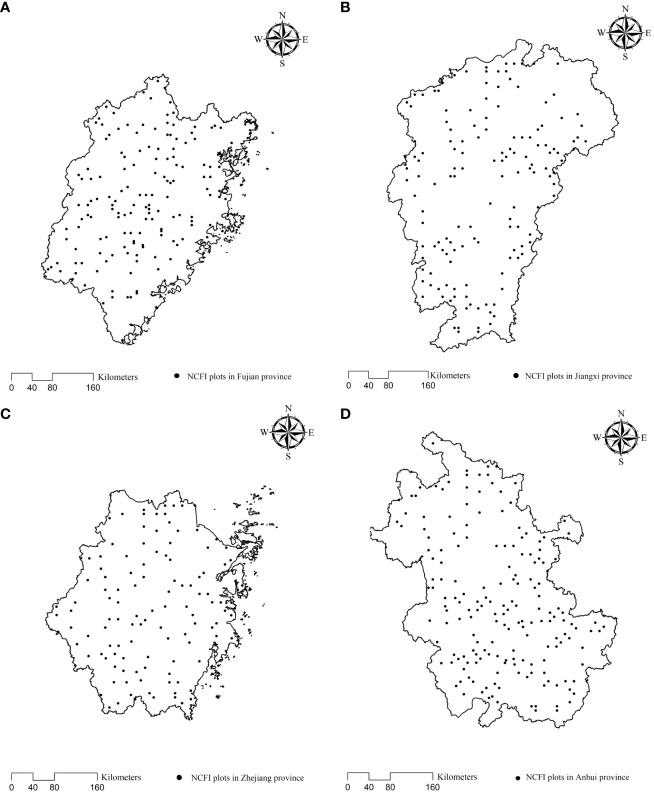
Distribution of National Forest Inventory (NFI) plots in Fujian **(A)**, Jiangxi **(B)**, Zhejiang **(C)**, and Anhui **(D)** provinces.

National Forest Inventory plots of Fujian (a 667 m^2^ square), Jiangxi (a 667 m^2^ square), Zhejiang (an 800 m^2^ square), and Anhui (a 667 m^2^ square) Provinces were established. The sample area was converted to units of hectares. The plots for this study were selected using the following criteria: (1) pure Chinese fir stands were unthinned, and seedlings were planted; (2) the volume of Chinese firs comprised more than 30% of the total stand; (3) the number of trees per hectare (*TPH*) continuously decreased with the quadratic mean diameter (*QMD*) every 5 years of observations according to the Andrews et al. (2018) method; (4) environmental disturbance (e.g., windthrow, pest, and disease) and artificial damage (e.g., illegal thinning) were absent; and (5) the regeneration of broad-leaved seedlings was natural. The selected plots were inevitably under self-thinning conditions. A total of 591 plots were selected, including 149, 146, 117, and 179 study sites in Fujian, Jiangxi, Zhejiang, and Anhui provinces, respectively (**Figure 2**).

### 2.2 Measurements and calculations

Measurements were taken from all trees above 5 cm in diameter at breast height (*DBH*) in every permanent plot; data collected included tree species, *DBH*, tree height, and tree status (living/dead). *DBH* was measured to the nearest 1 mm with diameter tape at a tree height of 1.3 m. Tree heights were measured (to the nearest 10 cm) with a Vertex (Haglöf Sweden). The recorded plot factors included soil type, latitude, longitude, altitude, slope aspect, slope position, slope gradient, soil thickness, soil texture, and humus thickness.

The number of living trees (*DBH*≥5 cm) per hectare is a basic index of stand density. *QMD* is a basic index that reflects the mean tree size in sample plots. It was calculated using (Equation 1).


(1) $$QMD=\sqrt{\frac{1}{N}\sum_{i=1}^{N}{\left(DBH\right)}_{i}^{2}}$$

where ${\left(DBH\right)}_{i}$ is the *DBH* of the *i* tree, and *N* is the total number of trees in the sample plot.

A site index (*SI*), representing site conditions, was estimated for each plot using the average height of the most dominant trees (95^th^ percentile and above) per hectare at the reference age of 20 years.

To determine tree species dominance in Chinese fir-dominated mixed forests, we calculated the ratio of the Chinese fir stand basal area to the total stand basal area (*BA*_Chinese fir_ */BA*_total_) in sample plots (Sun et al., 2011). When *BA*_Chinese fir_/*BA*_total_<0.5, the broad-leaved small trees were dominant in stands; however, when *BA*_Chinese fir_/*BA*_total_ *>*0.5, Chinese fir were dominant in stands. In addition, *BA*_Chinese fir_ */BA*_total_ */*(1- *BA*_Chinese fir_ */BA*_total_) was used to describe the mixing proportion (*MP*) of each plot.

In this study, we used latitude to represent precipitation and temperature in different provinces to clarify the effect of climate changes on maximum size–density relationships in Chinese fir-dominated mixed forests.

The correlation was calculated using the “corrplot” package in R software (R Core Team, 2023), and the “ggplot” package was used to plot the results. Stochastic frontier analysis was performed using FRONTIER 4.1 (Coelli, 1996). The adjusted coefficient of determination (R_adj_^2^), root mean square error (RMSE), and Pearson correlation coefficient between estimated and observed values (r) were used as metrics to assess the efficacy of the model’s fit.

### 2.3 Maximum size–density relationships

Bi et al. (2000) introduced stochastic frontier analysis to estimate maximum size–density relationships of even-aged *Pinus radiata* stands. The generalized expression (Equation 2) of the self-thinning relationship is as follows (Bi et al., 2000):


(2) $$Y=AX_{1}^{\beta_{1}}X_{2}^{\beta_{2}}\cdots X_{k}^{\beta_{k}}e^{\vartheta }e^{-\mu }$$

where *Y* is the observed value of the dependent variable related to stand growth; X_1_,…X_k_ are the independent variables affecting stand growth; *β*_1_, *β*_2_…*β_k_* are the exponents of the independent variables; *A* is the parameter of the model to be estimated; and $e^{\vartheta }\text{ and }e^{-\mu }$ are the two error exponents. Taking the logarithm, the expression (Equation 2) becomes (Equation 3) (Bi et al., 2000):


(3) lnY=α+βlnX+ε

where α=lnA  , *β* is a vector of parameters. The error term, ε=ϑ−μ , is a compound random variable with two components, and each is assumed to be independently and identically distributed across observations.

Bi (2001) incorporated site productivity in the stochastic frontier function for a generalized expression (Equation 4) of the self-thinning relationship:


(4) $$Y=AN^{\beta_{1}}S^{\beta_{2}}e^{\vartheta }e^{-\mu }$$

where *N* is the number of trees, *S* is the relative site index, *A* is the intercept parameter, and *β*_1_ and *β*_2_ are parameters. The variables ϑ and *μ* are two random variables.

Taking the logarithm, the expression (Equation 4) becomes (Equation 5) (Bi, 2001):


(5) $$lnY=\alpha +\beta_{1}lnN+\beta_{2}lnS+\varepsilon$$

where α=lnA , the error term ϵ is a compound random variable, ε=ϑ−μ . We introduced the mixing proportion and latitude into the model in expression (5) to analyze their effects on the number of trees, the slope and intercept of the self-thinning relationship, and the correlations between variables and determine the maximum stand density of the mixed-species forests. The model (Equation 6) can be expressed as follows (Kimsey et al., 2019):


(6) $$\ln\left(TPH\right)=\alpha +\beta_{1}\text{ln}\left(QMD\right)+\beta_{2}((\frac{BA_{Chinese\;fir}}{BA_{total}})/\left(1-\frac{BA_{Chinese\;fir}}{BA_{total}}\right))+\beta_{3}\ln\left(latitude\right)+\beta_{4}\left(SI\right)+\varepsilon$$

where *TPH* is the number of living trees per hectare; QMD is the quadratic mean diameter of each plot; $(\frac{BA_{Chinese\;fir}}{BA_{total}})/\left(1-\frac{BA_{Chinese\;fir}}{BA_{total}}\right)$ represents the mixing proportion; latitude represents climate changes (temperature and precipitation); *SI* is the site index; *α* is the intercept parameter; $\beta_{1}$ , $\beta_{2}$ , $\beta_{3}$ , and $\beta_{4}$ are parameters; and ϵ is the error term.

These models (Equation 7), with similar structures but different variable effects, resulted in a single, parsimonious model adaptable to each species that could be generally stated as (Kimsey et al., 2019):


(7) $$\begin{array}{l} \ln(TPH)=\\ \left\{ \begin{array}{l} \alpha +\beta_{1}\ln(QMD)\;\mathrm{PP1}\\ \alpha +\beta_{1}\ln(QMD)+\beta_{2}((\frac{{BA}^{\;}Chinese\;fir}{{BA}^{\;}total})/(1-\frac{{\;}^{BA}Chinese\;fir}{{\;}^{BA}total}))\;PP2\\ \alpha +\beta_{1}\ln(QMD)+\beta_{2}((\frac{{\;}^{BA}Chinese\;fir}{{\;}^{BA}total})/(1-\frac{{\;}^{BA}Chinese\;fir}{{\;}^{BA}total}))+\beta_{3}\ln(latitude)\;PP3\\ \alpha +\beta_{1}\ln(QMD)+\beta_{2}((\frac{{\;}^{BA}Chinese\;fir}{{\;}^{BA}total})/(1-\frac{{\;}^{BA}Chinese\;fir}{{\;}^{BA}total}))+\beta_{3}\ln(latitude)+\beta_{4}(\mathrm{SI})\;PP4 \end{array}\right. \end{array}$$

where the *TPH* for any given species is a function of *QMD* (PP1), species basal area proportion $(\frac{BA_{Chinese\;fir}}{BA_{total}})/\left(1-\frac{BA_{Chinese\;fir}}{BA_{total}}\right)$ (PP2), latitude (PP3), and *SI* (PP4). Species models (PP1–PP4) are additive in nature to clarify relative sequential effects on *TPH*.

## 3 Results

### 3.1 Changes in the self-thinning slope and intercept in Chinese fir-dominated mixed forests

On the log-log scale, the number of living trees per hectare (*TPH*) was negatively correlated with the quadratic mean diameter (*QMD*) of Chinese fir-dominated mixed forests in the different provinces (**Table 2**). That is, as *QMD* increased, *TPH* decreased by 1.605. Compared with the theoretical self-thinning slope of -1.605 in an even-aged pure stand, the estimated slope did not deviate from -1.605, albeit its trajectory was flatter for Chinese fir-dominated mixed forests (PP1 in **Table 2**).

**Table 2:** Summary of maximum stand density stochastic frontier model parameters of Chinese fir-dominated mixed forests in Fujian, Jiangxi, Zhejiang, and Anhui provinces.

Model	Intercept	Slope	*BA/(1-BA)*	ln*latitude*	*SI*	R_adj_^2^	RMSE	r
Fujian province								
PP1	7.827[7.085, 8.570]	-1.284[-1.682, -0.886]				0.9615	0.3437	0.4399
PP2	7.829[7.081, 8.576]	-1.268[-1.675, -0.861]	0.002[-0.016, 0.020]			0.9675	0.3436	0.4401
PP3	20.123[-10.020, 30.266]	-1.287[-1.689, -0.885]	0.003[-0.016, 0.022]	-1.152[-3.150, 0.846]		0.9725	0.3436	0.4401
PP4	25.521[-6.977, 58.019]	-1.291[-1.698, -0.883]	0.006[-0.013, 0.025]	-1.158[-3.307, 0.992]	-0.030[-0.053, -0.006]	0.9830	0.3401	0.4584
Jiangxi province								
PP1	8.567[8.144, 8.990]	-1.480[-1.643, -1.316]				0.7080	0.1610	0.6596
PP2	8.575[8.156, 8.993]	-1.431[-1.692, -1.170]	0.037[0.011, 0.062]			0.8905	0.1335	0.6514
PP3	26.735[2.218, 51.251]	-1.454[-1.662, -1.245]	0.027[-0.001, 0.055]	-1.267[-2.857, 0.322]		0.9168	0.1252	0.6742
PP4	27.457[3.769, 51.144]	-1.471[-1.635, -1.307]	0.028[-0.001, 0.056]	-1.220[-2.864, 0.424]	-0.013[-0.033, 0.007]	0.9341	0.1182	0.7414
Zhejiang province								
PP1	8.890[7.751, 10.030]	-1.394[-1.872, -0.915]				0.9148	0.2422	0.5944
PP2	9.172[7.850, 10.494]	-1.297[-1.773, -0.821]	0.051[0.031, 0.072]			0.9175	0.2369	0.6291
PP3	20.928[4.476, 37.380]	-1.535[-2.014, -1.056]	0.044[0.024, 0.065]	-1.847[-2.990, -0.704]		0.9232	0.2352	0.6342
PP4	25.242[6.338, 44.146]	-1.588[-2.146, -1.031]	0.051[0.030, 0.072]	-1.152[-2.332, 0.028]	-0.041[-0.075, -0.007]	0.9261	0.2352	0.6277
Anhui province								
PP1	8.945[8.164, 9.727]	-1.563[-1.872, -1.254]				0.9386	0.2654	0.7908
PP2	9.036[8.237, 9.835]	-1.549[-1.865, -1.233]	0.049[0.021, 0.077]			0.9537	0.2562	0.8004
PP3	21.905[6.924, 36.887]	-1.597[-1.909, -1.285]	0.043[0.015, 0.072]	-0.862[-1.857, 0.133]		0.9658	0.2539	0.8031
PP4	24.059[8.246, 39.873]	-1.696[-2.076, -1.316]	0.044[0.016, 0.073]	-1.000[-2.047, 0.047]	-0.015[-0.046, 0.017]	0.9695	0.2532	0.8028

R_adj_^2^, adjusted coefficient of determination; RMSE, root mean squared error; r, Pearson coefficient of correlation between estimated and observed values.

Maximum size–density relationships remained consistent as the mixing proportion, latitude, and site conditions increased. Compared with the self-thinning trajectory for *TPH*~*QMD* (PP1 in **Table 2**), the self-thinning slope was flat, even when the mixing proportion factor was included (PP2 in **Table 2**). It became steeper when latitude was included (PP3 in **Table 2**), and even more so when the site condition factor was included (PP4 in **Table 2**).

In the PP4 model, the slopes of the maximum size–density relationships were shallower in Fujian, Jiangxi, and Zhejiang provinces and steeper in Anhui Province than in even-aged pure stands, yet the slopes of the maximum size-density relationships for all provinces (PP4 in **Table 2**) were consistent with Reineke’s assumed universal slope of -1.605 at the 95% confidence level, with no deviation from the slope of the self-thinning relationship in even-aged pure stands. The inclusion of interaction terms (e.g., ln(*QMD*)*SI) did not significantly affect the model intercept or slope coefficients (Kimsey et al., 2019). Interaction terms were not included in the PP4 model.

**Figure 3:**
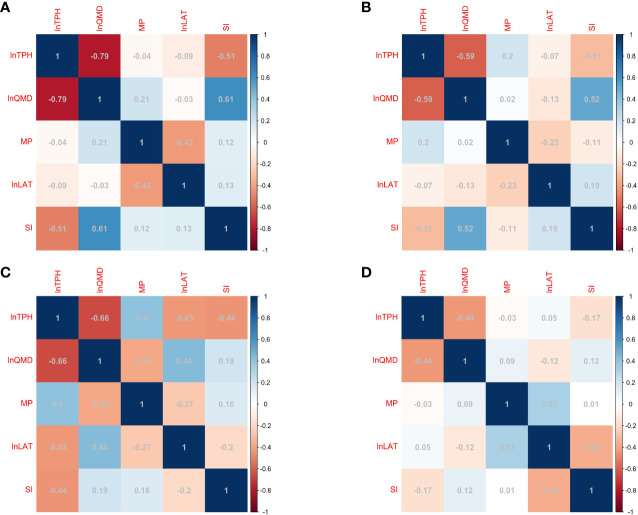
Correlations among ln*TPH* and independent variables in Fujian **(A)**, Jiangxi **(B)**, Zhejiang **(C)**, and Anhui **(D)** provinces.

The correlation coefficients were lower between the mixing proportion (or latitude) and *TPH* than between the other variables (e.g*.*, *QMD* and *SI*) (**Figure 3**). However, the number of remaining Chinese firs and naturally renewed broad-leaved tree species in different plots varied with the mixing proportion. Moreover, temperature and precipitation conditions at different latitudes in southern China vary, and this affects the self-thinning of Chinese fir and the regeneration of broad-leaved tree species. The addition of the mixing proportion and latitude variables to the model led to an increase in the R_adj_^2^ of the model and a decrease in the RMSE (**Table 2**). Therefore, the addition of the mixing proportion and latitude variables is important for clarifying maximum size–density relationships in Chinese fir-dominated mixed forests.

### 3.2 Factors affecting maximum size–density relationships in Chinese fir-dominated mixed forests

Mortality rates declined as the mixing proportion of Chinese fir-dominated mixed forests increased (PP2 in **Table 2**). In the PP4 model, the mortality rate of Chinese fir-dominated mixed forests decreased by 0.006, 0.028, 0.051, and 0.044 in Fujian, Jiangxi, Zhejiang, and Anhui provinces, respectively, for every unit increase in mixing proportion at the same latitude and site conditions (**Table 2**).

The PP3 model showed that Chinese fir-dominated mixed forests had a higher carrying capacity in lower-latitude provinces than in higher-latitude provinces (**Table 2**). The mortality rate of Chinese fir-dominated mixed forests increased by 1.158, 1.220, 1.152, and 1.000 in Fujian, Jiangxi, Zhejiang, and Anhui provinces for every unit increase in latitude at the same mixing proportion and site conditions (PP4 in **Table 2**). As the latitude increased (from Fujian Province to Anhui Province), the slope of the maximum size–density relationship gradually became steeper, and the number of living trees per hectare changed in parallel (PP3 in **Table 2**).

Forest site had a negative effect on maximum size–density relationships (PP4 in **Table 2**). Regardless of the mixing proportion and latitude, the mortality rate of Chinese fir-dominated mixed forests in Fujian, Jiangxi, Zhejiang, and Anhui provinces increased by 0.030, 0.013, 0.041, and 0.015 for every unit increase in site conditions (PP4 in **Table 2**).

**Figure 4:**
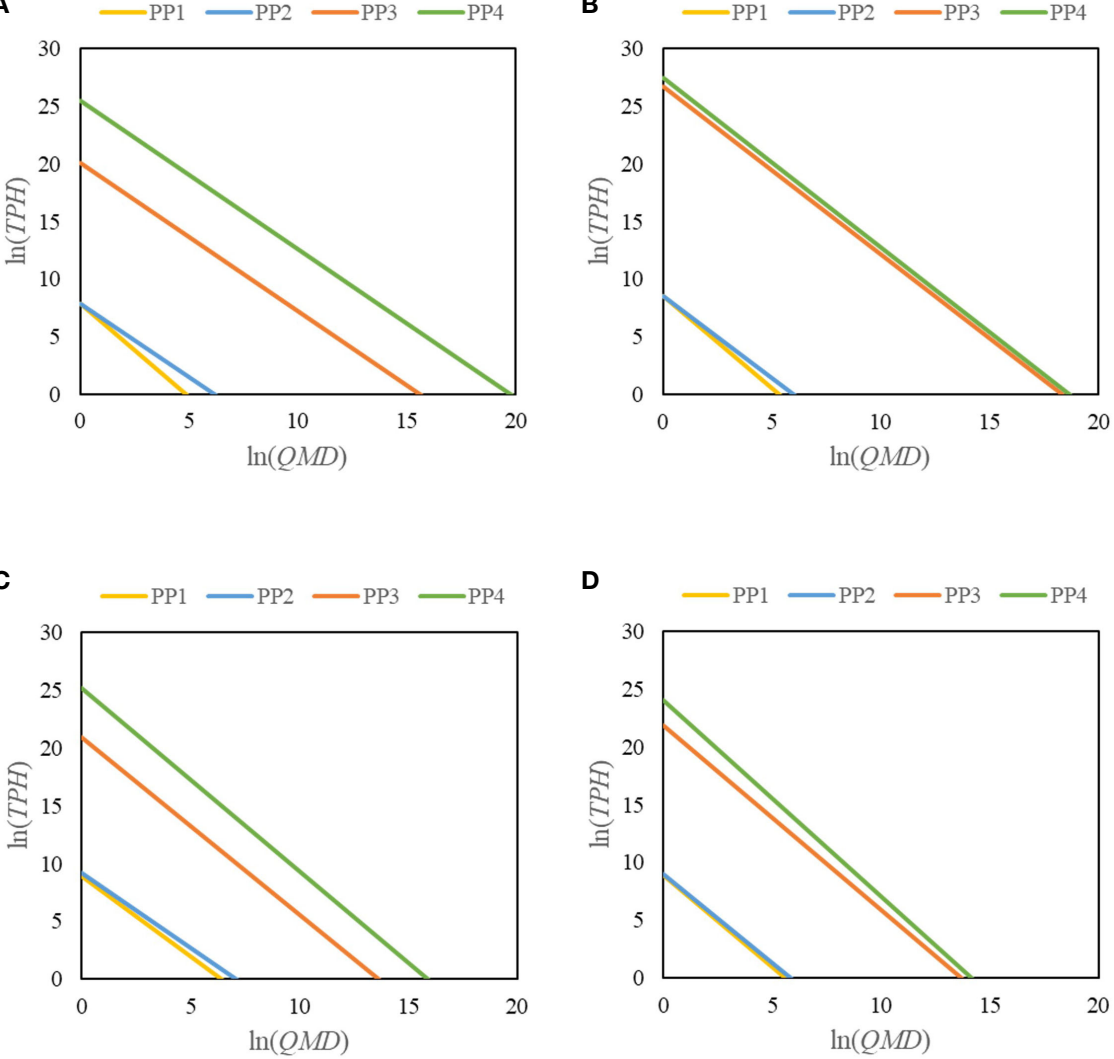
Changes in the maximum size–density relationship when the variables mixing proportion (PP2), latitude (PP3), and site conditions (PP4) in Fujian **(A)**, Jiangxi **(B)**, Zhejiang **(C)**, and Anhui **(D)** provinces were included relative to the original model (PP1).

The magnitudes of the effects of mixing proportion, latitude, and site variables on maximum size–density relationships varied (**Figure 4**). Latitude had a stronger negative effect on maximum size–density relationships than mixing proportion and site conditions. The slope of the maximum size–density relationship of the Chinese fir-dominated mixed forests did not deviate from the slope of the self-thinning relationship of the pure forests.

## 4 Discussion

### 4.1 The slope of the maximum size–density relationship of Chinese fir-dominated mixed forests

The ecological carrying capacity for any given forest is driven by size–density relationships (Shaw, 2006). The difference in the slope of the limiting relationship is an indication of differences in the potential of a stand to withstand an understocked site and crowding conditions. The slope of the maximum size–density relationship of Chinese fir-dominated mixed forests was flatter than that of even-aged pure Chinese fir stands (PP1 in **Table 2**), and changes in the number of living trees per hectare were mainly driven by the appearance of natural broadleaved tree species and the death of a few Chinese fir trees (Sun et al., 2011). Flatter slopes indicate a greater capacity for a stand to withstand low-density conditions as diameter growth increases, and a poor ability of trees to survive crowding conditions (Gadow, 1986).

Our study showed that the slope of the maximum size–density relationships of the PP4 model in each province was -1.605 (PP4 in **Table 2**), which did not differ from the slope of the self-thinning trajectory in the even-aged pure stands. The characteristics of the Chinese fir-dominated mixed forests, including the uneven age of the stands and the presence of multiple layers, especially the vertical crown layers, were consistent with the self-thinning trajectory. The lack of a difference in the slope of the maximum size–density relationships and the slope of the self-thinning trajectory of even-aged pure stands can be explained by a dynamic negative feedback mechanism that regulates stand density; the negative feedback between trees reflects the increasing dominance of the upper Chinese fir crown layer and the continuous death of lower broadleaved tree species during the self-thinning process. The absence of a slope difference can also be explained by the observation that light was the main factor directly affecting the self-thinning trajectory of mixed forests with high stand densities (Morris, 1999).

In this study, Chinese fir-dominated mixed forests comprised even-aged pure Chinese fir stands and natural tree species. Chinese fir occupied the upper crown layer, and naturally regenerated broadleaved tree species were located in the secondary crown layer. The main competitive interactions between Chinese fir and natural tree species are associated with differences in tree size. As mixed forests age, the growth space and habitat resources required by trees increase continuously. Asymmetric competition among trees intensifies (Xue and Hagihara, 1999; Stoll et al., 2002; Ogawa, 2005), and the interactions between adjacent individuals are stronger than interactions between non-adjacent individuals. The larger Chinese fir trees outcompete the smaller broadleaved tree species for light; this eventually leads to deviation from the light compensation point and the death of broadleaved trees (Weiner and Thomas, 1986), which further enhances the dominance of Chinese fir.

### 4.2 The effect of species mixtures on maximum size–density relationships in Chinese fir-dominated mixed forests

The death of trees in mixed forests is caused by a combination of the lack of light and limited growth space (Long et al., 2022). When even-aged pure stands of Chinese fir are converted to Chinese fir-dominated mixed forests, self-thinning of Chinese fir occurs, which involves the death of less dominant Chinese fir trees and the formation of canopy gaps. Canopy gaps allowed some broadleaved tree species, especially evergreen shade-tolerant tree species (e.g., *Schima superba* Gardner & Champ, *Cinnamomum camphora* (Linn) Presl, and *Phoebe zhennan* S. K. Lee & F. N. Wei), to grow in the secondary layer.

Chinese fir is a light-demanding tree that occupies the upper canopy when other shade-tolerant tree species are present, and shade-tolerant broadleaved tree species are located in the lower canopy. Niche differentiation of the vertical space can improve the overall light use efficiency of forests (Dănescu et al., 2016). The canopy interception of Chinese fir decreased during the self-thinning process, given that the precipitation in the atmosphere can penetrate through the forest layer and directly into the soil via canopy gaps, which enhances the supply of soil nutrients and water.

### 4.3 The effect of latitude on the maximum size–density relationships of Chinese fir-dominated mixed forests

Temperature and rainfall play a significant role in modifying both the slope and intercept of the maximum size–density relationship in a given species (Kweon and Comeau, 2017; Andrews et al., 2018; de Prado et al., 2020). Latitudinal differences are closely related to climate conditions, which have implications for the growth and distribution of tree species. The growth of trees is mainly limited by temperature in high-latitude areas. By contrast, the effect of water availability on the growth of trees becomes increasingly important as the latitude decreases (Babst et al., 2013). Chinese fir is a shade-intolerant tree species with a low growth rate, and self-thinning is delayed in high-latitude areas. Decreases in temperature impede the regeneration of broadleaved tree species in the lower canopy and reduce stand density (Wu, 1984).

When water availability is not limited, the survival probability of tree species is high in warm areas. Some studies have shown that warm, dry forest sites have lower stand densities than cool, moist forest sites, which have higher numbers of shade-tolerant species (Weiskittel et al., 2009; Ducey et al., 2017). However, warmer conditions can enhance Chinese fir growth and decrease tree mortality (Wu, 1984). In addition, warm climates likely promote more rapid increases in photosynthetic activity than in respiration rates, which enhances the carbon assimilation rate (Way and Oren, 2010; Lines et al., 2012).

### 4.4 The effect of site conditions on the maximum size–density relationships of Chinese fir-dominated mixed forests

Site conditions do not affect the maximum size–density relationships of mixed forests, but they have positive effects on average tree size growth as forests develop; stand density decreases as forests experience competition-induced mortality (Harms et al., 2000; Pittman and Turnblom, 2003; VanderSchaaf and Burkhart, 2008). Canopy closure can occur rapidly in forests, and competition among trees can be particularly intense at fertile sites, which can result in canopy gaps (Yan et al., 2021). However, living trees, especially small broadleaved tree species, can rapidly colonize canopy gaps at fertile forest sites.

As site conditions increased, the number of living trees per hectare decreased (PP4 in **Table 2**). Trees with high fertility allocated more biomass to crown growth, which accelerated the competition among tree species for light, and this resulted in the death of competitively inferior tree species (Morris, 2002; Hautier et al., 2009; Campoe et al., 2013). The asymmetric competition for light is intensified at fertile sites, which results in the death of small Chinese fir trees. Moreover, the differences in tree size between Chinese fir and broadleaved tree species are particularly pronounced at highly fertile sites.

## 5 Conclusion

The results of this study revealed the maximum size–density relationships of Chinese fir-dominated mixed forests using National Forest Inventory data from four provinces in southern China, as well as the effects of growth, species mixing, latitude, and site conditions on the self-thinning trajectory using stochastic frontier analysis. We found that (1) The slope of the maximum size–density relationship of Chinese fir-dominated mixed forests on the log-log scale moved upward and became flat; (2) the slope of the maximum size–density relationship of Chinese fir-dominated mixed forests did not deviate from Reineke’s assumed universal slope of -1.605; and (3) mixing proportion had a positive effect on maximum size–density relationships, and latitude and site conditions had negative effects on maximum size–density relationships.

## Data availability statement

The original contributions presented in the study are included in the article/supplementary material. Further inquiries can be directed to the corresponding author.

## Author contributions

YH: Writing – original draft, Formal analysis, Visualization. BW: Investigation, Supervision, Writing – review & editing. HS: Conceptualization, Funding acquisition, Methodology, Project administration, Supervision, Writing – review & editing.
